# Investigation of Salt Tolerance Mechanisms Across a Root Developmental Gradient in Almond Rootstocks

**DOI:** 10.3389/fpls.2020.595055

**Published:** 2021-01-05

**Authors:** Yuhang Shao, Yukun Cheng, Hongguang Pang, Mingqin Chang, Fang He, Minmin Wang, Destiny J. Davis, Shuxiao Zhang, Oliver Betz, Chuck Fleck, Tingbo Dai, Shahab Madahhosseini, Thomas Wilkop, Judy Jernstedt, Georgia Drakakaki

**Affiliations:** ^1^Department of Plant Sciences, University of California, Davis, Davis, CA, United States; ^2^Key Laboratory of Crop Physiology Ecology and Production Management of Ministry of Agriculture, Nanjing Agricultural University, Nanjing, China; ^3^Triticeae Research Institute, Sichuan Agricultural University, Chengdu, China; ^4^College of Horticulture, Hebei Agricultural University, Baoding, China; ^5^College of Forestry, Sichuan Agricultural University, Chengdu, China; ^6^Sierra Gold Nurseries, Yuba City, CA, United States; ^7^Genetic and Plant Production Department, Vali-e-Asr University of Rafsanjan, Rafsanjan, Iran; ^8^Light Microscopy Core, University of Kentucky, Lexington, KY, United States

**Keywords:** almond rootstocks, ion exclusion, vacuolar sequestration, suberin, lignin, endodermis, exodermis, salinity tolerance

## Abstract

The intensive use of groundwater in agriculture under the current climate conditions leads to acceleration of soil salinization. Given that almond is a salt-sensitive crop, selection of salt-tolerant rootstocks can help maintain productivity under salinity stress. Selection for tolerant rootstocks at an early growth stage can reduce the investment of time and resources. However, salinity-sensitive markers and salinity tolerance mechanisms of almond species to assist this selection process are largely unknown. We established a microscopy-based approach to investigate mechanisms of stress tolerance in and identified cellular, root anatomical, and molecular traits associated with rootstocks exhibiting salt tolerance. We characterized three almond rootstocks: Empyrean-1 (E1), Controller-5 (C5), and Krymsk-86 (K86). Based on cellular and molecular evidence, our results show that E1 has a higher capacity for salt exclusion by a combination of upregulating ion transporter expression and enhanced deposition of suberin and lignin in the root apoplastic barriers, exodermis, and endodermis, in response to salt stress. Expression analyses revealed differential regulation of cation transporters, stress signaling, and biopolymer synthesis genes in the different rootstocks. This foundational study reveals the mechanisms of salinity tolerance in almond rootstocks from cellular and structural perspectives across a root developmental gradient and provides insights for future screens targeting stress response.

## Highlights

This study reveals potential mechanisms of salt stress tolerance in almond *via* ion exclusion and sequestration and cellular barrier differentiation across a root developmental gradient.

## Introduction

Soil salinization is one of the major environmental factors limiting agricultural productivity. Both natural and human activities have exacerbated this critical problem over the years (Rengasamy, 2002; Munns and Tester, 2008), resulting in a decrease in agricultural productivity in the affected areas. Worldwide almond production is a rapidly growing sector, with a 45% global increase in production from 2017 to 2018.^1^ However, the low salinity tolerance of almond plants is a significant hurdle negatively affecting almond production (Zrig et al., 2011; Kaundal et al., 2019). Therefore, selection of rootstocks with improved salinity tolerance is required to maintain the current production rate despite the trend of increasing soil salinity.

Plant roots are characterized by high developmental plasticity, facilitating adaptation to adverse environments (Liu et al., 2015). The root responds to salinity stress *via* inhibition of root growth, as a result of a decrease in the amount of cell expansion in the root elongation zone (Zhong and Läuchli, 1993; West et al., 2004; Jiang et al., 2017). Root adaptation to salinity stress involves development changes of the meristematic zone at the root tip, which is the most developmentally active region and one that plays a lead role in determining root system architecture (Julkowska et al., 2017). Salt stress leads to a reduction of root meristem size, coinciding with a shift in the ratio of lateral root to main root growth (Duan et al., 2013; Geng et al., 2013). Therefore, the different developmental layers in roots – the meristematic zone, the elongation zone, and the maturation zone – are expected to show different responses to salt stress. Currently, there is limited knowledge on salt tolerance mechanisms in a way that corresponds to developmental stages, especially in woody nut crops.

Known mechanisms of salinity tolerance include salt exclusion, vacuolar ion sequestration, and salt transportation (Munns and Tester, 2008; Zhang et al., 2010). Glasshouse experiments of citrus species indicated rootstock varieties that were able to exclude sodium ions (Na^+^) or chloride ions (Cl^−^) to minimize ion accumulation in the leaves (Sykes, 1992), a crucial feature in evaluating salt tolerance in citrus (Hussain et al., 2012).

Na^+^ sequestration is one of the well-studied mechanisms in salinity tolerance (Apse et al., 1999; Roy et al., 2014; Wu et al., 2019). In woody plants such as citrus, the vacuoles of root cortical parenchyma cells can sequester Na^+^ under salinity stress. Their ability to sequester salt in the root has been associated with salt reduction in the shoots, contributing to salinity tolerance (Gonzalez et al., 2012; Martínez-Alcántara et al., 2015). The ion antiporter encoded by *NHX1/2* (Blumwald and Poole, 1985; Adabnejad et al., 2015), which is involved in vacuolar sodium sequestration, has been associated with increased salinity tolerance in diverse species such as tomato, mung bean, wheat, and rice (Moghaieb et al., 2014; Kumar et al., 2017; Zeng et al., 2018). In contrast to wide studies in diverse species, the capacity of vacuolar sodium sequestration in almond rootstocks, especially the difference between high salinity tolerance and low salinity tolerance genotypes, is currently unknown.

One of the key families of genes involved in salinity stress response is the *SALT OVERLY SENSITIVE* (SOS). Originally identified through a forward genetics study in *Arabidopsis thaliana*, *SOS1* has been shown to encode a Na^+^/H^+^ antiporter at the plasma membrane responsible for efflux of salt from the cytoplasm to the apoplast. SOS1 activity is induced by salt stress and is mediated by the calcium-activated SOS2–SOS3 protein kinase complex (Shi et al., 2000; Qiu et al., 2002). The SOS genes also coordinate with high-affinity K^+^ channels (HKT; Yang and Guo, 2018). HKT, together with the low-affinity K^+^ channels (AKT), help maintain ion homeostasis by controlling sodium and potassium flux at the plasma membrane. HKT and AKT therefore play a role in regulating Na^+^ entry into the roots by selectively transporting K^+^ under salt stress (Rus et al., 2001; Apse and Blumwald, 2007). A recent study showed that PpHKT1, the HKT1 ortholog in almond rootstock “Nemaguard” (*Prunus persica* × *Prunus davidiana*), is able to rescue *athkt1* salt-sensitive phenotype (Kaundal et al., 2019), suggesting a conserved role in almond.

Apoplastic barriers regulate solute and water transport in roots. The endodermis and the exodermis are two cell layers located in the innermost and outermost border of the root cortex, respectively. Both cell layers contain two components that make up the root apoplastic barrier: (1) the Casparian strip enriched in lignin and (2) the suberin lamellae (Enstone et al., 2002).

Lignin is primarily responsible for the diffusion barrier located at the Casparian strip of the endodermis (Naseer et al., 2012). Its deposition along the wall at the exodermis and the endodermis forces water and solutes to pass from the less regulated apoplastic route into the more controlled symplastic transport route (Naseer et al., 2012). Therefore, the deposition of lignin and the development of the Casparian strip in response to salt stress have been of significant interest in salinity tolerance studies (Yeo et al., 1999; Chen et al., 2018). Previous studies of 4-Coumarate 3-hydroxylase (C3H), an enzyme that catalyzes the formation of phenolic acids in lignin synthesis (Goujon et al., 2003), indicated that C3H may contribute to salt stress tolerance by regulating the production of reactive oxygen species and improving oxidase activity in broccoli plants (Jiang et al., 2017). Similarly, over-expression of the *C3H* gene improves salt tolerance in tobacco (Guo et al., 2009) and tamarisk (Wang et al., 2019).

The suberin lamellae is deposited in the endodermis and exodermis in more mature root zones, following the deposition of the Casparian strip, and plays an important role in the bidirectional regulation of solute transportation (Enstone et al., 2002; Barberon 2017; Doblas et al., 2017). The suberin lamellae is also developmentally plastic and highly responsive to nutrient (Barberon et al., 2016) and abiotic stress (Reinhardt and Rost, 1995; Tylová et al., 2017). In *Oryza sativa*, roots can increase the apoplastic barrier suberin deposition in response to salinity stress, which, in turn, reduces total sodium accumulation in shoots (Krishnamurthy et al., 2009). The expression of lignin and suberin biosynthesis genes (*C3H*, *ASFT1*, and *KCS1*; Rains et al., 2018) is highly upregulated in response to osmotic stress in barley (Kreszies et al., 2019). Among these genes, KCS1 is a 3-ketoacyl-CoA synthase responsible for fatty acid chain elongation in suberin biosynthesis (Todd et al., 1999). ASFT1, a feruloyl-CoA transferase, catalyzes the transfer of the phenolic head towards the acyl-chain in suberin biosynthesis and has been implicated in salinity tolerance in *Arabidopsis* (Zhang et al., 2020).

In addition to salt ion transport, sequestration, and minimizing salt ion entry *via* apoplastic barriers, plants counter the osmotic stress induced by high soil salinity with the use of osmolytes, such as proline (Gupta and Huang, 2014; Yang and Guo, 2018). P5CS1 is a delta1-pyrroline-5-carboxylate synthase that catalyzes the rate-limiting step in the biosynthesis of proline, whose cellular levels are associated with osmotic homeostasis (Maghsoudi et al., 2018). Because leaf chlorosis is often associated with salinity stress (Husain et al., 2003), the long-distance coordination of systemic responses is necessary. Phospholipid signaling under salinity stress involves SAL1, also known as *FRY1*, which represents a point of crosstalk between stress homeostasis and stress detoxification pathways (Zhu, 2002). Currently, it is unknown whether proline-dependent osmotic protection or retrograde chloroplast signaling is part of the salinity tolerance mechanism in almonds.

The main goal of this study was to characterize the salinity stress responses in three almond rootstocks. The study was based on the hypothesis that the first defense under salinity takes place in the root system and that sodium uptake and sequestration and apoplastic barrier differentiation contribute to salinity response. The expression of genes involved in related pathways can reflect these changes. Our study has altogether provided new insights into the cellular salt tolerance mechanisms of almond plants, generated a reference map for further root analysis, and identified differences in salinity stress response across a root developmental gradient.

## Materials and Methods

### Growth Conditions and Treatments

Three genotypes, including Empyrean®-1(E1; *Prunus persica* × *Prunus davidiana*), Controller-5 (C5; *Prunus salicina* × *Prunus persica*), and Krymsk®-86 (K86; *Prunus cerasifera* × *Prunus persica*), were used for the experiments. Clonally propagated rootstocks were transferred from the rooting medium (supplied by Sierra Gold Nurseries, Yuba City, CA, USA) to soil and grown for 2 months in controlled environmental chambers with 55–65% humidity at 28°C and with light intensity of 800 μM/cm^2^/s. Subsequently, the plants were watered with 50 ml half-strength Hoagland solution containing 0, 50, and 150 mM NaCl per pot per day. Leaf weight, root weight, and root length were measured at harvest after 13 days of treatment. The shoots and roots were imaged at harvest.

### Root Tip Harvest and Sections

After treating the plants for either 3 or 13 days, the root tips were harvested from three plants per genotype per treatment for staining. The root tips were harvested in 1-cm sections from the distal root tip to 3 cm above the root tip and immobilized in 5% agarose using sectioning blocks as mold (Electron Microscopy Sciences, 70180-AS). Next, the agarose-embedded root tips were sectioned at 100-μm thickness using a Vibratome (Vibratome 1,000 Plus Sectioning System, Richmond, IL, USA). The sections were transferred to an incubation buffer (20 mM MOPS, 0.5 mM CaSO_4_, and 200 mM sorbitol) and kept at 4°C for up to 1 week.

### Sodium and Fluorescein Diacetate Staining

The vibratome sections were transferred into CoroNa Green sodium staining solution. Fifty micrograms of CoroNa Green reagent (ThermoFisher, Waltham, MA, USA) was dissolved in 100 μl dimethyl sulfoxide and further diluted to 0.1 mM working solution in the aforementioned incubation buffer to constitute the sodium staining solution. The samples were incubated overnight in sodium staining solution at room temperature in the dark prior to imaging with a Zeiss confocal microscope (Zeiss LSM 700).

Because both CoroNa Green signal and sodium sequestration require live cells for dye processing and high salinity stress may induce cell death, we analyzed the cell viability of the root sections after the 3‐ and 13-day salt treatments. We used fluorescein diacetate (FDA) as a viability stain because its fluorescence depends on cell membrane integrity (Jones et al., 2016). A stock solution of 0.2% FDA (w/v; Sigma-Aldrich, F7378) was made in acetone and then diluted to 4 μg/ml in incubation buffer. Immediately following sectioning, fresh samples were transferred to the staining solution and incubated in the dark at 22°C for 20 min. The FDA-stained samples were imaged with a Zeiss confocal microscope (Zeiss LSM 700) within 30 min.

### Suberin and Lignin Staining

To investigate apoplastic barriers in the salinity tolerance of almond rootstocks, we first observed suberin deposition with Nile red, a lipophilic stain (Ursache et al., 2018). Stock solutions of 0.1% (w/v) Nile red were made in acetone and subsequently diluted to 2 μg/ml in 75% glycerol (in double-deionized water) to form the staining solution. Fresh sections were incubated in the staining solution at room temperature in the dark for 30 min. The sections preserved in the incubation buffer were rinsed twice with deionized water before staining. We measured the signal intensity at the endodermis and the exodermis and recorded the number of suberized cells in the endodermis to quantitatively analyze the suberin differences between rootstocks and across the root developmental gradient upon salt stress.

The dominant biopolymer of cellular barriers, lignin, was assessed using an established protocol based on Fuchsin Basic staining using the ClearSee sample preparation (Ursache et al., 2018). Our adapted ClearSee solution contains 10% (w/v) xylitol (Sigma-Aldrich), 15% (w/v) sodium deoxycholate (Sigma-Aldrich), and 25% (w/v) urea (Sigma-Aldrich) to reduce chlorophyll auto-fluorescence (Kurihara et al., 2015). Then, 0.2% (w/v) Fuchsin Basic was dissolved in ClearSee solution for the lignin staining solution. The sections were stained in basic fuchsin staining solution overnight at room temperature in the dark. Before imaging, the stained samples were de-stained with ClearSee solution overnight at room temperature in the dark.

### Confocal Laser Scanning Microscopy

LSM700 (Carl Zeiss), Axio Imager 2 was used for imaging. A 488-nm laser was used for FDA‐ and CoroNa-stained samples (at 2 and 10.58% laser power, respectively), while a 555-nm laser was used to image the suberin‐ and lignin-stained samples (40 and 6% power, respectively). Emissions were collected over a wavelength range of 493 to 800 nm for FDA and CoroNa Green and 560–800 nm for Fuchsin Basic and Nile red. All images were collected using the Plan-Apochromat × 20 0.8 M27 objective. The pinhole setting was 1.32 U for FDA, 1.40 U for sodium, 1.54 U for lignin, and 1.96 U for suberin. The Z-series of the root tip sections were imaged at 2-μm intervals. Zen 3.1 (Zeiss) software was used for imaging and image export.

### Gene Expression Analysis

Candidate gene expression levels in the root tips were determined by qPCR. After 3 days of salt treatment, 3 cm of whole root tips were collected for all genotypes, rinsed with deionized water, and flash-frozen in liquid nitrogen. Total RNA was extracted with the RNeasy Plant Mini Kit (Qiagen, Germantown, MD, USA). cDNA was synthesized using the High Capacity cDNA Reverse Transcription kit (ThermoScientific, Waltham, MA, USA). The relative quantification method was used for analyzing the abundance of transcripts (Schmittgen and Livak, 2008). *ACTIN2* was used as a reference gene. The coding sequences of *Arabidopsis NHX1*, *NHX2*, *SOS1*, *SOS2*, *SOS3*, *P5CS1*, *SAL1*, *HKT1*, and *AKT1* (Shi et al., 2000; Rus et al., 2001; Qiu et al., 2002; Apse and Blumwald, 2007; Bassil et al., 2011; Estavillo et al., 2011; Barragan et al., 2012; Maghsoudi et al., 2018) and barley *ASFT1*, *KCS1*, and *C3H* (Kreszies et al., 2019) were used in a homolog search using BLAST against the *Prunus persica* transcriptome. The primers targeting these genes are listed in Supplementary Table 1. Quantitative analysis was performed by real-time RT-PCR using the PowerSYBR Green PCR Master Mix (Thermo Scientific).

### Data Analysis and Figure Assembly

**Figure 1:**
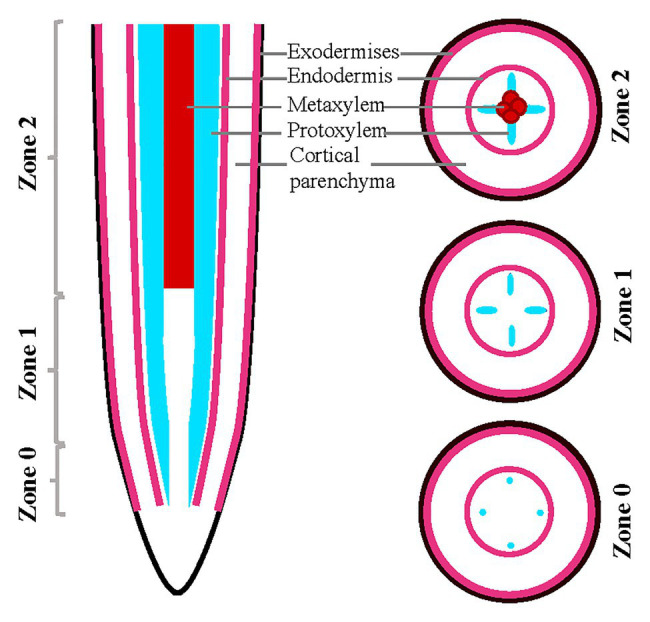
Schematic representation of root sections in almonds roots. Schematic of almond root in longitudinal and transverse sections indicating the designation of zones utilized in this analysis, staged based on xylem developmental anatomy.

All the images obtained with the Zeiss confocal microscope (Zeiss LSM 700) were analyzed with ImageJ. Analysis across the root developmental gradient was performed by dividing the root tips into three developmental zones staged by tracking xylem development as follows: Z0, defined by the initiation of protoxylem largely overlapping with the meristematic zone; Z1, defined by a fully developed protoxylem; and Z2, defined by the presence of metaxylem and being the more mature zone (Figure 1).

Statistical analyses were performed using Microsoft Excel 2016 (Microsoft, Redmond, WA, USA) and SPSS ver. 11.0 (SPSS Inc., Chicago, IL, USA). Means were computed from a minimum of three biological replicates and subjected to analysis of variance (ANOVA) to determine statistical significance. Two-way ANOVA was performed for plant growth data, and three-way ANOVA was performed for the image quantification data. Means were compared using Duncan’s multiple-comparison tests (SPSS Inc., Chicago, IL, USA). Microsoft Excel was used to perform *t*-tests and generate graphs. The relative fluorescence intensity of sodium, FDA, suberin, and lignin staining was obtained after subtracting the unstained controls. All figures were assembled using Affinity Designer (Serif Europe Ltd., UK).

## Results

### Phenotypic Responses of Different Rootstocks in Salinity Stress

**Figure 2:**
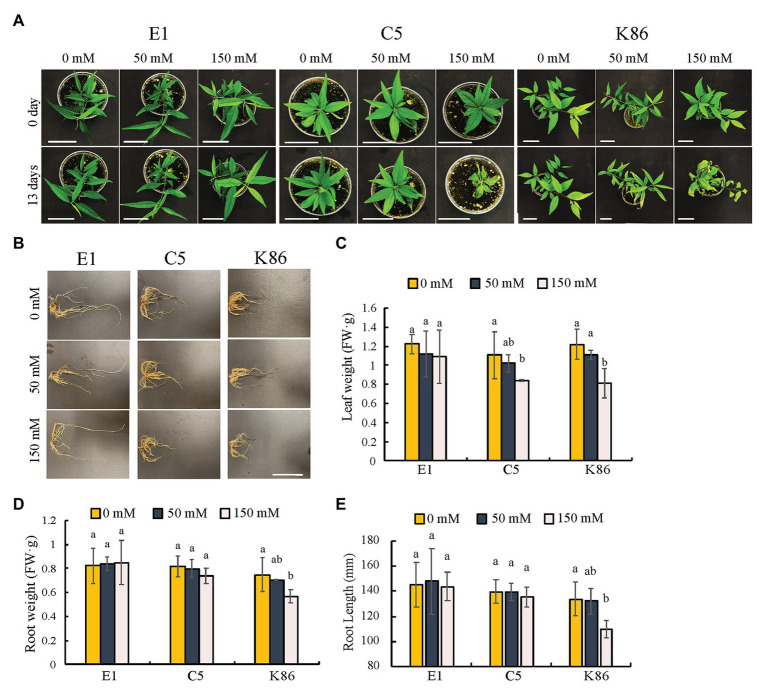
Phenotypic comparisons of E1, C5, and K86 under salt treatment. Phenotype comparisons between E1, C5, and K86 plants before and after treatment with 0, 50, and 150 mM NaCl for 13 days. **(A)** Representative images of whole plants of E1, C5, and K86. Scale bar, 5 cm. **(B)** Representative root images of E1, C5, and K86. Scale bar, 5 cm. **(C)** Quantification of leaf weight, **(D)** root weight, and **(E)** root length suggests that E1 has superior salt tolerance compared to C5 and K86. Values are means ± SE (*n* = 5). Different lowercase letters indicate a significant difference at *p* < 0.05, two-way ANOVA.

In order to evaluate the salinity response of the three selected rootstocks, we first assessed the shoot phenotypes. During the first week of treatment, there were no discernable differences between the different NaCl concentrations and the no-treatment controls. However, genotype-specific salinity stress responses were observed by day 13 under 150-mM-NaCl treatment (Figure 2). Empyrean-1 (E1) showed minimal chlorosis on the young leaves compared to the untreated controls (Figure 2A). In contrast to E1, leaf senescence and wilting were apparent in Controller-5 (C5) and Krymsk-86 (K86) after 13 days of 150-mM-NaCl treatment (Figure 2A). Leaf weight analysis showed that there was no significant decrease in E1 after either 50‐ or 150-mM treatment, while both C5 and K86 showed a significant decrease of leaf weight under 150-mM-NaCl treatment (Figure 2C).

We next examined the overall root phenotype (Figure 2B). Under control conditions, there was no significant difference in root length or weight between the three genotypes, and neither E1 nor C5 showed a significant decrease in root length and weight even after the 150-mM-salt treatment (Figures 2D,E). K86 showed the strongest response to salinity among the three genotypes, with a significant reduction in root mass after the 150-mM-NaCl treatment (Figures 2B,D). Our results altogether show that E1 outperformed the other two genotypes based on the salinity stress symptoms.

### Sodium Transport and Sequestration in Almond Rootstocks

**Figure 3:**
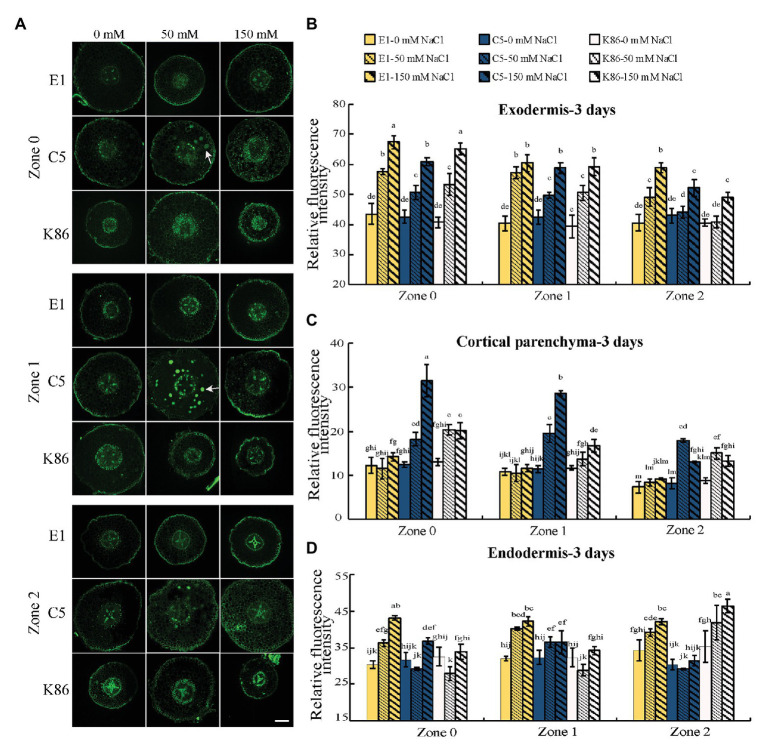
E1 shows lower sodium accumulation under salinity stress compared with C5 and K86. **(A)** Representative images of CoroNa Green staining in different root developmental zones of E1, C5, and K86 treated with 0, 50, and 150 mM NaCl for 3 days. White arrows highlight sodium vacuolar localization in C5. Scale bars, 100 μm. **(B)** Quantification of sodium signal in the exodermis, **(C)** cortical parenchyma, and **(D)** endodermis shows lower fluorescent signal, etc. Values are means ± SE (*n* = 15). Different lowercase letters indicate significant differences at *p* < 0.05, three-way ANOVA.

In order to gain insight into the mechanisms contributing to salinity tolerance, we visualized the localization of sodium across a developmental gradient at the root tip, where most salt absorption takes place. C5 and K86 showed a higher sodium signal intensity compared to E1 in cortical tissues under 50‐ and 150-mM-NaCl treatments for all zones (Z0–Z2; Figure 3A). Quantification of the sodium staining signal confirmed our cellular observations, with higher staining in the cortical parenchyma tissue of C5 and K86, compared to E1, and with C5 showing the highest sodium signal under the 150-mM-NaCl treatment (Figure 3C). This suggested a higher amount of sodium in the roots of K86 and C5 compared to E1 and that E1 may be more efficient in minimizing salt ion entry.

Thus, we next focused on the sodium signal at the apoplastic barriers, the exodermis, and the endodermis under salt treatment. Interestingly, the sodium staining intensity at the apoplastic barrier showed a reverse trend of that for cortical parenchyma staining in the exodermis. In the exodermis, E1 clearly showed the strongest sodium staining signal of the three genotypes for all zones (Figures 3A,B). In the endodermis, E1 showed the strongest sodium signal in Z0 and Z1, while K86 showed the weakest signal at these zones for both salt treatments. However, at Z2, K86 showed the highest endodermis sodium signal at both 50‐ and 150-mM treatments (Figures 3A,D). Our data altogether indicate that apoplastic cell barriers may contribute differently to salinity tolerance based on the root developmental stage and that the role of the exodermis and the endodermis may be differentially regulated under salinity stress.

We observed a high sodium signal in the cortical parenchyma cells of C5 rootstocks and what appears to be vacuolar sodium signal. Because vacuolar sequestration of sodium has previously been shown in other species (Gonzalez et al., 2012; Roy et al., 2014), we also analyzed the vacuolar localization of sodium across genotypes and treatments. There was a noticeable increase in sodium accumulation in the vacuoles of cortical parenchyma tissue in C5 under 50-mM-NaCl treatment in all zones compared to the untreated control (Figure 3A). However, vacuolar sequestration was not observed following 150-mM-NaCl treatment, suggesting that C5 likely possesses a limited capacity for sodium sequestration. No obvious vacuolar sodium sequestration was observed in either E1 or K86 under these experimental conditions (Figure 3A).

Our results altogether show that E1 exhibits the least amount of sodium accumulation in root cortical parenchyma cells for all zones under both salt treatments, while C5 has limited potential in vacuolar sodium sequestration under relatively lower salt (50 mM) treatment.

### Cell Viability Staining of the Root Meristematic Cells of Three Rootstocks Upon Salt Treatment

**Figure 4:**
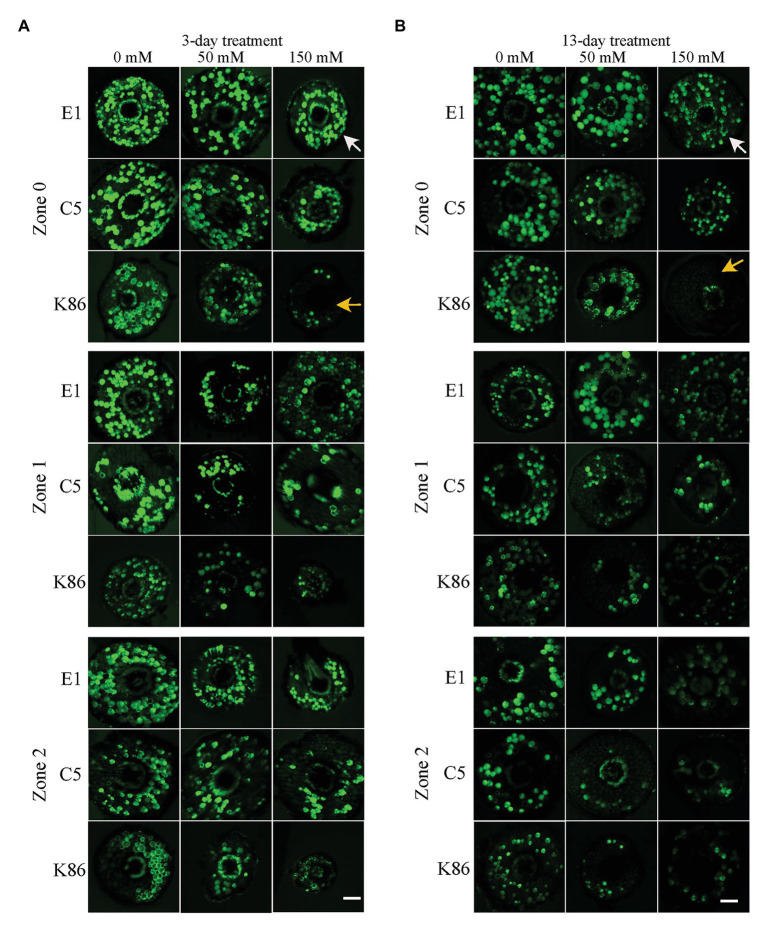
Cell viability across a root developmental gradient in salt-stressed rootstocks. Cell viability analysis in E1, C5, and K86 treated with 0, 50, and 150 mM NaCl shows decreased viability with increased length of treatment **(A)** and **(B)**. Representative images of fluorescein diacetate (FDA) staining in the root of E1, C5, and K86 under a 3-day **(A)** and a 13-day **(B)** salt treatment. White arrows highlight live cells in E1. Yellow arrows indicate a reduction in the number of live cells in K86. Scale bar, 100 μm. Representative images of *n* = 15.

**Figure 5:**
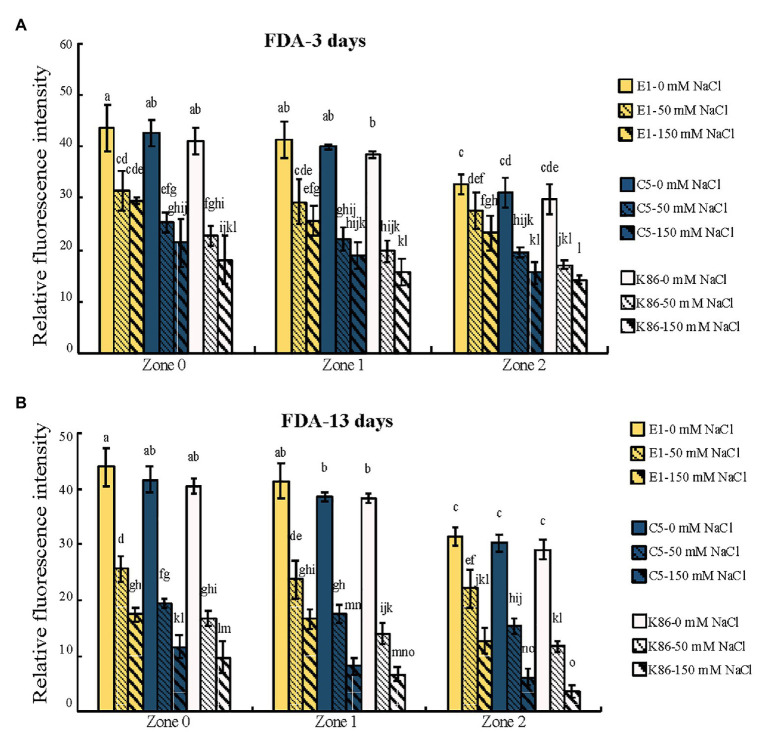
Quantification of cell viability across the root developmental gradient of E1, C5, and K86. Quantification of cell viability indicates that E1 has the highest number of viable cells under salt treatments. Fluorescence quantification of fluorescein diacetate (FDA) staining in the roots of E1, C5, and K86 treated with 0, 50, and 150 mM NaCl for 3 days **(A)** and 13 days **(B)**. Analysis shows a reduction in cell viability concomitant with increased salt concentration and duration of treatment. Values are means ± SE (*n* = 15). Different lowercase letters indicate a significant difference at *p* < 0.05, three-way ANOVA.

In order to verify the biological relevance of our results, we analyzed the cell viability of the root sections after the 3‐ and 13-day salt treatments using FDA staining. As shown in Figures 4, 5, both 50‐ and 150-mM-NaCl treatments led to a decrease in cell viability, with the 150-mM-NaCl treatment causing the greatest reduction. Under control conditions, Z2 showed a lower FDA fluorescence than Z0 for all genotypes (Figure 5). There was no significant difference among the three genotypes without NaCl treatment (Figures 4, 5). However, under both 3‐ and 13-day salt treatments, E1 showed the highest number of viable cells compared to C5 and K86 under both 50‐ and 150-mM-NaCl treatments (Figures 5A,B). Since FDA indicates the integrity and the activity of cells, it is likely that, among the three genotypes, E1 roots are able to maintain the highest number of viable cells under salinity stress. Therefore, based on our cell viability analysis, our results suggest that E1 has the highest and K86 the lowest salt tolerance.

### Suberin Deposition in Response to Salt Treatment

**Figure 6:**
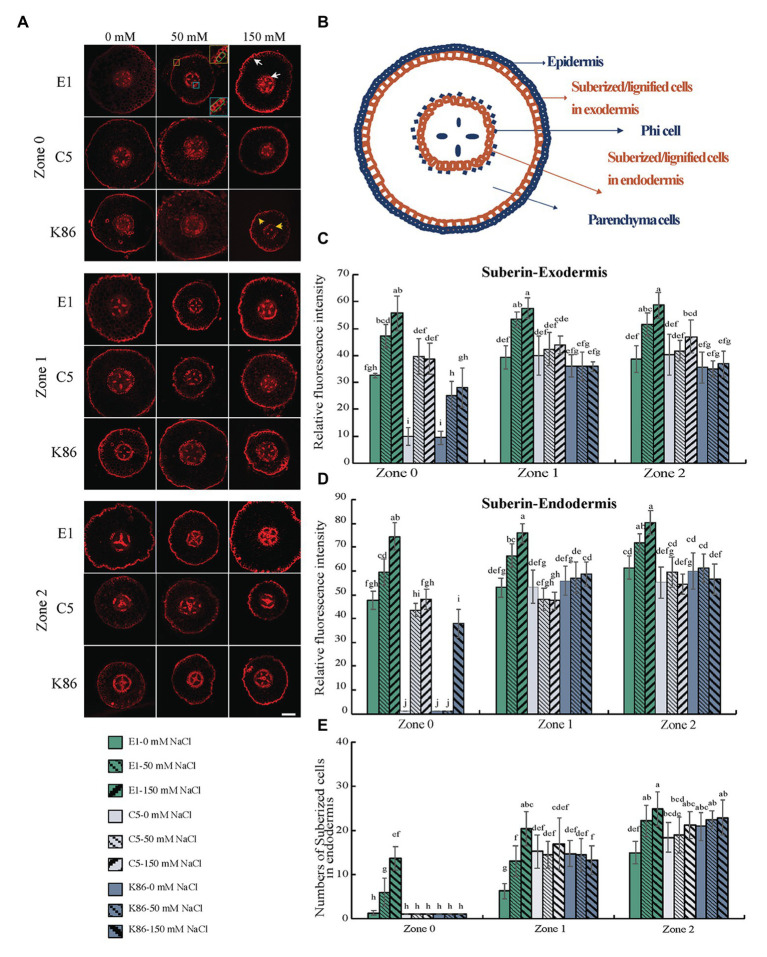
Suberin deposition across a developmental gradient in almond rootstocks. Suberin deposition across a developmental gradient in E1, C5, and K86 treated with 0, 50, and 150 mM NaCl for 3 days. **(A)** Representative images of suberin staining in the root of E1, C5, and K86 across different developmental zones. Scale bar, 100 μm. Yellow box highlights the exodermis; cells highlighted by a green box are quantified for fluorescence intensity. Blue box highlights the endodermis; cells highlighted by a green box are quantified for fluorescence intensity. White arrows indicate the suberized cells in exodermis and endodermis in E1. Yellow arrows indicate the reduced number of suberized cells in K86. **(B)** Representative diagrams of almond root in transverse section indicating the location of apoplastic barriers and phi cells. Quantification of suberin staining in exodermis **(C)** and endodermis **(D)** of E1, C5, and K86 indicates higher suberization and increased salinity stress response in E1. **(E)** Quantification of suberized cell number in endodermis of E1, C5, and K86. Values are means ± SE (*n* = 15). Lowercase letters indicate a significant difference at *p* < 0.05, three-way ANOVA.

To gain insight into the potential salt exclusion mechanism at apoplastic barriers in almond rootstocks, we observed suberin deposition between the different genotypes under salt treatment (Figure 6A). We measured the signal intensity at the endodermis and exodermis (Figure 6B) and recorded the number of suberized cells in the endodermis to quantitatively analyze the suberin differences between rootstocks and across the root developmental gradient. As expected, the deposition of suberin increased with the increasing maturity of the developmental zones examined (Figure 6A). E1 showed the greatest increase in suberin deposition in the exodermis in response to all salt treatments across all zones (Figure 6C). In contrast, C5 showed a significant increase between control and 50-mM treatment in Z0, but no further increase between 50‐ and 150-mM treatments, and only minimal increases in Z1 and Z2 in response to both salt treatments (Figure 6C). K86 showed the lowest exodermis suberin signal of the three genotypes. Although it did show an increase in suberization in response to rising salt concentrations in Z0, it failed to respond to salt treatment in Z1 and Z2 (Figure 6C).

Under control conditions, minimal to no suberin signal was detected in the endodermis of C5 or K86 at Z0. In contrast, E1 showed suberin deposition as early as Z0, even under control conditions (Figures 6A,D). In addition, E1 showed the highest suberization signal for each treatment in all zones (Figure 6D). In contrast, C5 showed increased suberin deposition under 50-mM-NaCl treatment compared to the control in Z0, yet no significant increase in Z1 and Z2 (Figure 6D). Similarly, K86 increased suberin deposition only in Z0 upon 150-mM treatment, while no significant changes were observed in Z1 and Z2 (Figure 6D). These data suggest a higher response to salinity stress in Z0, reflecting the plasticity of this developmental zone.

We then analyzed the number of suberized cells in the endodermis (Figure 6E) and showed that, in all three zones, E1 had the highest number of suberized cells, with the increase in the number of suberized cells corresponding to an increase in sodium concentration. However, the number of suberized cells did not increase in the other genotypes in Z1 and only minimally in Z2. Our data altogether show that, among the three genotypes, E1 is the most responsive to salinity stress with respect to suberin deposition, both in the exodermis and the endodermis across all developmental zones.

### Lignin Deposition in Response to Salt Treatment

**Figure 7:**
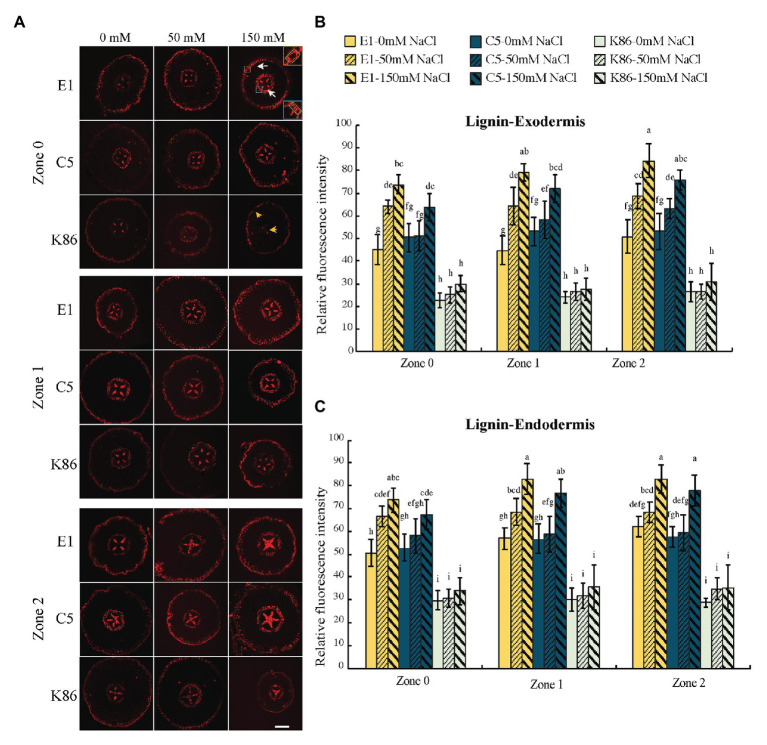
Lignin deposition across a developmental gradient in almond rootstocks. Lignin deposition across the developmental gradient in E1, C5, and K86 treated with 0, 50, and 150 mM NaCl for 3 days. **(A)** Representative images of lignin staining in the roots of E1, C5, and K86. Scale bar, 100 μm. Yellow box highlights the exodermis; cells highlighted by a green box are quantified for fluorescence intensity. Blue box highlights the endodermis; cells highlighted by a green box are quantified for fluorescence intensity. White arrows indicate the lignified cells in exodermis and endodermis in E1. Yellow arrows indicate that K86 shows a reduction in the number of lignified cells. Quantification of lignin staining in exodermis **(B)** and endodermis **(C)** of E1, C5, and K86 shows increased lignification in response to salinity stress in both E1 and C5, but not in K86. Values are means ± SE (*n* = 15). Lowercase letters indicate a significant difference at *p* < 0.05, three-way ANOVA.

We examined lignin, the dominant polymer of cellular barriers, both in the exodermis and the endodermis of the root sections (Figure 7A). In control conditions, E1 and C5 showed stronger signals compared to K86 for all zones in both the exodermis and the endodermis (Figures 7B,C). Then, we analyzed the intensity of lignin signal by zone for all treatments in both the exodermis and the endodermis (Figures 7B,C). There was a correlation of lignin staining intensity with salt concentration in both the exodermis and the endodermis of E1 in all developmental zones. Although the same trend was also observed in C5 exodermis and endodermis, only the 150-mM-NaCl-treated samples of C5 showed a significant difference compared to the control, in both Z1 and Z2 (Figures 7B,C). K86, in contrast, showed the weakest lignin signal among the three genotypes, even under control conditions. Unlike E1 and C5, K86 did not show any increase in lignin deposition in any of the salt treatments in either the exodermis or the endodermis, indicating a lower responsiveness to salinity stress in apoplastic barrier development (Figures 7B,C). Notably, lignified phi cells, a cortical cell type hypothesized to contribute to salt ion uptake regulation (Fernandez-Garcia et al., 2009), were stained in some, but not all, sections in all three genotypes across all developmental zones (Figure 7A).

### Expression of Genes Associated With Rootstock Salt Tolerance

**Figure 8:**
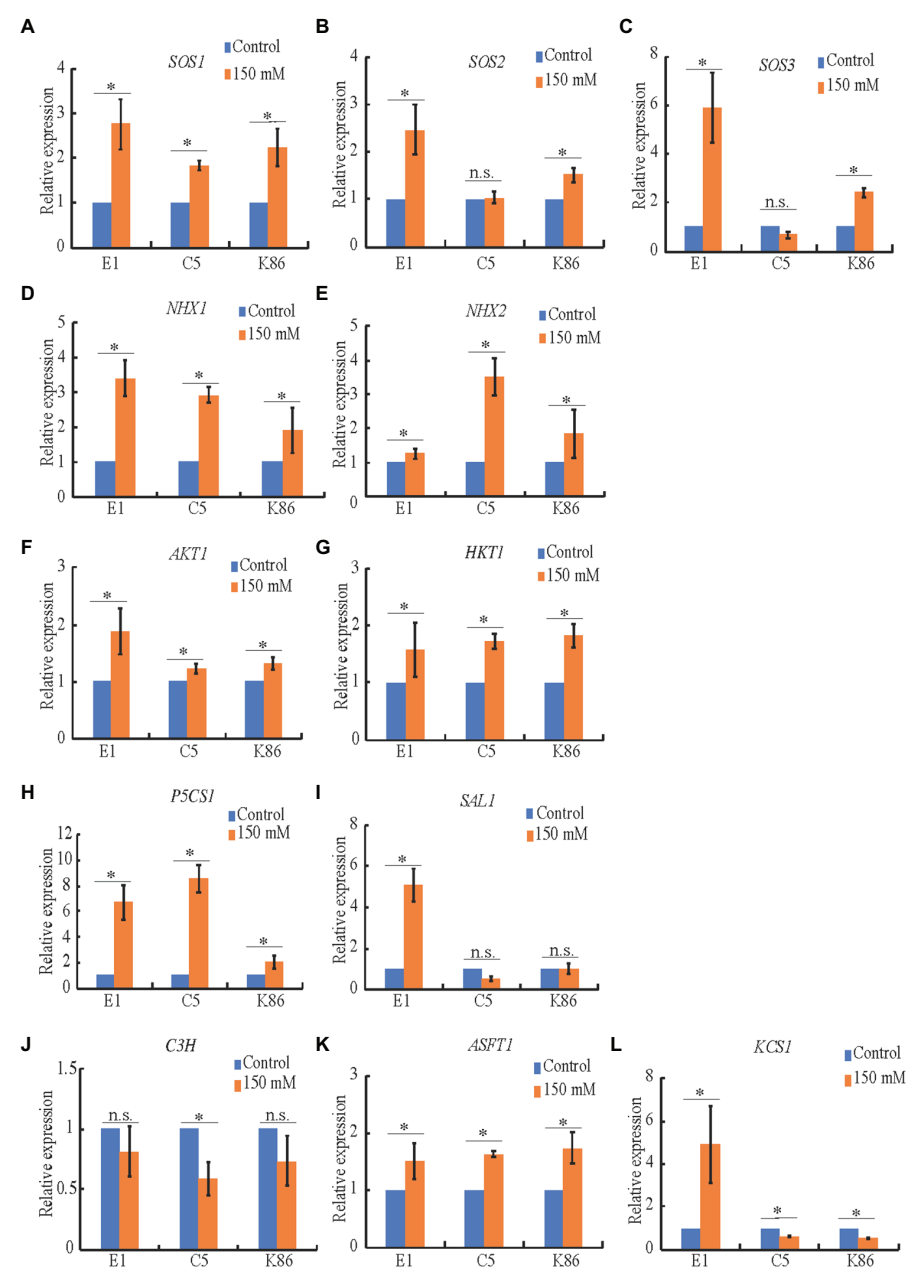
Expression analysis of candidate genes in salinity stress in almond rootstocks. RT-qPCR analysis of the expression level of candidate salinity stress genes in E1, C5, and K86 under control conditions and 150 mM NaCl treatment for 3 days. **(A-C)** *SOS* genes show a significant upregulation in salt-treated samples in E1. **(D,E)** Candidate genes involved in the sequestration of Na^+^ in vacuoles. *NHX1* is upregulated in all genotypes in response to salinity stress. *NHX2* is upregulated threefold in C5 plants. **(F,G)** Candidate genes involved in Na^+^ transport are upregulated in response to salinity stress, with *AKT1* showing a higher upregulation in E1. **(H)** Proline biosynthesis is upregulated in response to salinity stress in all three genotypes, with a weaker response in K86. **(I)** The abiotic stress signaling pathway is highly upregulated in E1, but not C5 or K86. **(J-L)** Candidate genes involved in suberin biosynthesis show more significant upregulation compared to lignin biosynthesis. *KCS1* shows a significant upregulation of expression in E1 compared to downregulation in C5 and K86. Values are means ± SE (*n* = 12). ^*^p < 0.05.

To better understand the effect of salt treatments on ion transporters, sodium sequestration, signaling, and biopolymer synthesis, we performed expression analysis of candidate genes in almond, i.e., homologs to salinity-responsive genes characterized in other species. Quantitative real-time PCR indicated that *SOS1* (Figure 8A), *NHX1* (Figure 8D), *HKT1* (Figure 8G), *P5CS1* (Figure 8H), and *ASFT1* (Figure 8K) were all upregulated upon salinity stress in the three tested genotypes, while *SOS2* and *SOS3* (Figures 8B,C), *AKT1* (Figure 8F), and *KCS1* (Figure 8L) were more highly upregulated in E1. This supports our hypothesis that E1 has an increased capacity to exclude salt, as the higher expression of *SOS2* (Figure 8B) and *SOS3* (Figure 8C) can lead to activation of the SOS1 antiporter to extrude sodium. NHX2 (Figure 8E), a vacuolar antiporter responsible for sodium sequestration into the vacuole, was dramatically upregulated in C5, corroborating sodium vacuolar sequestration (Figure 3A). *SAL1*, part of the retrograde abiotic stress signaling pathway from the chloroplast to the nucleus (Figure 8I), was only upregulated in E1, while *P5CS1*, involved in the synthesis of the osmotic stress-protectant proline (Figure 8H), was upregulated in both C5 and E1. This suggests that, while both *SAL1* and *P5CS1* may be involved in salinity response in almond rootstocks, *SAL1* may play a more critical role in salinity tolerance. *C3H*, part of the lignin biosynthesis pathway, did not significantly change upon salt treatment in E1 (Figure 8J), which is in line with similar observations in *Arabidopsis* (Chun et al., 2019). These gene expression patterns provide additional evidence for the molecular mechanisms behind salt tolerance in these almond rootstocks and valuable targets for future studies.

## Discussion

### Tolerant Rootstocks Exhibit Significant Phenotypic Evidence of Salinity Tolerance at the Early Growth Stage

The root is the first organ exposed to salinity stress and plays an important role in salt sensing and salt avoidance behavior (Galvan-Ampudia et al., 2013; Robbins et al., 2014; Koevoets et al., 2016; Julkowska et al., 2017). Salinity stress induces programmed cell death in the root and slows down cell activity, resulting in growth reduction and stress symptoms in the aerial parts of the plant (West et al., 2004; Koevoets et al., 2016). Exclusion and sequestration of salt ions by the root are known mechanisms that affect the ion concentration in shoot sap and the accumulation of toxic ions in the leaves (Tester and Davenport, 2003). This can be a result of more effective vacuolar compartmentalization of sodium ions, increased production of osmoprotectants such as proline, or more effective salt ion retrieval from shoot *via* ion transporter pathways. Because roots are the first defense layer under high salt conditions, we first assessed the three rootstocks for overall salt tolerance phenotypes in order to select rootstocks and analyze them in greater depth at the root level. Our root phenotypic analysis was used to generate hypotheses on possible tolerance mechanisms and inform our further molecular experiments.

An analysis of 3-month-old E1 rootstocks suggested multiple mechanisms of protection from salinity stress compared to other genotypes. E1 exhibited unaffected root growth and largely maintained root cell viability in salt stress compared to the control. In contrast, K86 had a significant reduction in both root length and weight, with minimal cell viability in the root cortex, in response to salinity stress. To draw conclusions on overall plant health and performance, we also observed salt stress symptoms in aerial tissues of all three rootstocks. While the aerial tissues of both C5 and K86 showed multiple symptoms of salt stress, including severe chlorosis and wilting shoot (Figure 2A), E1 exhibited only minor leaf chlorosis, verifying the salt tolerance of this genotype.

Our holistic analysis provided strong evidence for salinity tolerance in E1 over the two other rootstocks tested as well as provided both the experimental conditions and framework to better understand the mechanisms of salinity tolerance based on cellular and molecular responses in root tissues. Towards this end, we measured sodium uptake and sequestration across a root developmental gradient (Figure 3), while paying attention to root anatomical structures in almond that could contribute to salt tolerance.

### Vacuolar Sequestration in Response to Salinity Treatment Is Genotype Specific

Vacuolar sodium sequestration decreases sodium concentration in the cytoplasm and minimizes sodium transport to the shoot (Munns and Tester, 2008). Vacuolar sodium sequestration is a well-studied salt tolerance mechanism across several crop species (Blumwald et al., 2000; Barragan et al., 2012; Wu et al., 2019) but has not been assessed in almond. Of the three genotypes, C5 exhibited the most prominent vacuolar sodium sequestration effect. Vacuolar sodium signal was observed in C5 samples under the 50-mM-NaCl treatment but notably not in samples under the 150-mM-NaCl treatment (Figure 3). It is likely that higher concentrations of salt, as tested with the 150-mM-salt treatment, exceed the salt tolerance threshold and sequestration capacity of C5. Combined with our cell viability assay, it is plausible that high salt concentrations induce cell senescence that, in turn, hinders the cell’s ability to initiate or maintain vacuolar sequestration of NaCl (Figures 4, 5).

In contrast, E1 and K86 have no vacuolar sodium signal in any sample with or without NaCl treatment (Figure 3). The high salt tolerance phenotype of E1 roots suggests that alternative mechanisms for salinity tolerance, such as osmoprotectants and salt exclusion, may be in place and that vacuolar sequestration was not the primary strategy of tolerance under our treatment conditions and concentrations. Although a similar mechanistic explanation is possible for K86, the significantly diminished root growth and cell viability suggest that K86 may simply be far more salt sensitive than either C5 or E1 (Figures 4, 5). In this case, the cortical parenchyma cells of K86 are likely unable to sequester sodium due to a high rate of cell senescence and death (Figures 4, 5). Future studies with salt concentrations below 50 mM NaCl can provide further insights on the salt sensitivity of K86.

Vacuolar sodium sequestration is dependent on the activity of the vacuolar antiporter, NHX1/2, responsible for sodium sequestration (Barragan et al., 2012; Yamaguchi et al., 2013). In agreement with our vacuolar sodium staining results, we observed that *NHX2* was upregulated twofold in C5 in response to salt treatment but only slightly upregulated in E1 and K86 (Figure 8E). *NHX1* showed a twofold upregulation in both E1 and C5 (Figure 8E). This suggests that both genes may contribute to the sequestration observed in C5. However, because C5 exhibited a significantly higher level of vacuolar sequestration paired with significant upregulation of *NHX2*, we conclude that *NHX2* is likely the dominant antiporter for vacuolar sodium sequestration in almond rootstocks.

### Apoplastic Barriers Contribute to High Salinity Tolerance

The exodermis and the endodermis serve as two apoplastic barriers contributing to protection against abiotic stress, such as drought and salinity, by regulating the uptake and transport of water and ions from the soil (Seago et al., 2000; Enstone et al., 2002; Lux et al., 2004). The sodium localization data of E1 showed a significant accumulation in the cell walls of exodermis and endodermis (Figure 3), suggesting that sodium ion transport to the stele may be restricted by these two apoplastic barriers. Specifically, the enhancement of suberization at both exodermis and endodermis restricts ion transport from the rhizosphere to the stele by limiting apoplastic transport (Baxter et al., 2009). An anatomical analysis of suberin deposition showed that E1 exhibits a striking increase of suberin deposition at both exodermal and endodermal cells under salinity stress compared to the other two genotypes (Figure 6). Notably, the analysis across a root developmental gradient in E1 showed an increased number of suberized cells in the endodermis of the youngest developmental zone in E1 under 50‐ and 150-mM-salt stress. In contrast, C5 and K86 showed very few suberized cells in the endodermis of Z0 under both salt treatment conditions (Figure 6E). These data together suggest that E1 may be more efficient in salt exclusion in the root not only by increasing the amount but also by earlier suberin deposition, as it has been documented in other species under osmotic stress (Kreszies et al., 2019). Considering our vacuolar data, we reason that sufficient reduction in salt ion entry may be enough to reduce the sodium concentration in E1 cortical cells, such that vacuolar sequestration may not be necessary. Future analysis of salt ion concentration in both shoots and roots is necessary to determine if this is the case.

Our gene expression analysis confirmed the suberin detection experiments in that the 3-ketoacyl-CoA synthase, *KCS1*, involved in suberin and cutin biosynthesis (Mustroph and Bailey-Serres, 2010), is dramatically upregulated in E1, but not in C5 or K86 (Figure 8L). Notably, our root zone-dependent investigation method allowed us to examine the developmental time point of suberization initiation. E1 exhibited suberin lamellae deposition in zone 0 under mild and strong salt stress earlier than the other two more sensitive genotypes (C5 and K86; Figure 6). The early suberization in almond rootstocks, in contrast to the late suberin development in *Arabidopsis* (Naseer et al., 2012), indicates a mechanism for higher salinity tolerance in this woody species. We conclude that the combination of early suberization initiation and enhanced suberin lamellae deposition in E1 contributes to its high salinity tolerance by restricting sodium uptake.

Similar to suberin lamellae deposition, lignin deposition, particularly at the Casparian strip, is also critical for the regulation of solute transport at apoplastic barriers (Naseer et al., 2012). An anatomical analysis showed higher lignin deposition in E1 in both exodermal and endodermal cell walls compared to C5 and K86, which further supports our conclusion that limiting salt uptake contributed to salinity tolerance in E1. While *C3H*, a gene active in lignin biosynthesis, is upregulated in other species under osmotic stress (Barros et al., 2019; Kreszies et al., 2019), *C3H* did not significantly change upon salt treatment in E1 (Figure 8J). Considering that lignin biosynthesis genes are differentially regulated in response to abiotic stress in various species (Moura et al., 2010; Chun et al., 2019), the enhanced lignin deposition in E1 may be a result of other gene upregulations. Future work will assess which lignin biosynthesis genes are involved in the increased lignin in E1 under salt stress.

### Sodium Transporter Genes and Stress Signaling Genes Are Strongly Upregulated in Tolerant Rootstocks

In addition to the anatomical and cellular adaptations observed among the three rootstocks in response to salt stress, we also assessed the expression changes of key sodium transporter genes. The export of sodium at the plasma membrane is mediated by the ion transporter SOS1 and regulated by SOS2 and SOS3 (Qiu et al., 2002; Shi et al., 2002; Martínez-Alcántara et al., 2015), while overexpression of *SOS* genes has been shown to increase salt tolerance (Yang et al., 2009). The observed increased expression of all three *SOS* genes in E1 under salt stress (Figures 8A–C) strongly suggests that minimizing salt entry into cells, at the level of the plasma membrane, contributes to the salinity tolerance of E1, in addition to minimizing salt entry *via* apoplastic barriers. The sodium transporter HKTs are known to control the retrieval of sodium from the xylem and reduce sodium accumulation in shoots in response to salinity stress (Rus et al., 2001; Davenport et al., 2007; Kaundal et al., 2019; Khan et al., 2020). The upregulation of *HKT1* in all three rootstocks (Figure 8E) indicates that this transporter is conserved in salt stress response. The potassium channel AKT increases osmotic and drought tolerance by facilitating an increase in potassium uptake in rice and barely (Ahmad et al., 2016; Feng et al., 2020). We found that *AKT1* was upregulated significantly only in E1 (Figure 8D), suggesting that an increase in potassium uptake may be important in almond rootstock’s salt tolerance. The overall investigation of ion transport-associated genes indicates that ion balancing *via* exporting sodium and importing potassium is beneficial in almond salt tolerance. *SOS* and *AKT* genes therefore can be used as candidate reporters for the selection of salt-tolerant almond rootstocks.

SAL1, the phosphatase regulating the signal molecule 3'-polyadenosine 5'-phosphate level, is a negative regulator of drought tolerance in *Arabidopsis* (Wilson et al., 2009). The downregulation of *SAL1* in C5 (Figure 8I) upon salt stress suggests that SAL1 plays a similar role in C5. However, the fourfold upregulation of SAL1 in E1 (Figure 8I) indicates that a differential regulation of SAL1 might be depending on genotype, as it has been shown between two poplar species under drought stress (Street et al., 2006). This is likely due to the association of SAL1 with the altered root architecture in plants (Hirsch et al., 2011). This suggests that the root architectural features of E1 (Figure 2B) provide an advantage in response to salinity treatment compared to the other two genotypes.

*P5CS1*, involved in proline biosynthesis for drought adaptation (Muzammil et al., 2018), is significantly upregulated in E1 and C5 after salinity treatment, in contrast to K86 (Figure 8H), suggesting that protection against osmotic stress is a key feature of salt tolerance in almond rootstocks.

In summary, our molecular evidence showed that there are several mechanisms of salt tolerance at play in the almond rootstocks tested, including sodium ion transport, stress signaling, and osmotic balance. Among those, upregulation of genes involved in these pathways leads to increased salt tolerance, as shown with our E1 expression data.

## Conclusion

Our phenotypic and cellular analyses revealed that E1 achieves superior salt tolerance *via* multiple mechanisms focusing on the exclusion of sodium ions from the root vasculature. We observed evidence of apoplastic and symplastic salt exclusion through enhanced suberin lamellae and lignin deposition and upregulation of ion transporters. In contrast, our data suggest that vacuolar sequestration reaches a certain threshold beyond which other mechanisms are required for salinity tolerance. Additionally, a developmental gradient is critical for investigating salt stress response in the root. Our analysis across a root development gradient shows that apoplastic barriers, particularly in early developmental zones, play an important role in the salinity tolerance of these woody plant species. Our data suggest that K86 employs fewer salt tolerance strategies of the other two genotypes, which corroborates its relatively salt-sensitive phenotype.

In conclusion, our study addresses a long-standing limitation of identifying salt-tolerant rootstocks at an early growth stage. We report plantlet and cellular phenotypes and molecular evidence of salt tolerance, any of which can be used as markers in the selection of salt-tolerant genotypes. Additionally, this work provides a foundational study of a root anatomy map in almond rootstocks that can be applied beyond salinity to studies on other abiotic stresses. Furthermore, we have provided a new perspective on rootstock analysis and identified genes that can provide a basis of selection and modification of root characteristics towards salinity tolerance.

## Data Availability Statement

The original contributions presented in the study are included in the article/Supplementary Material, further inquiries can be directed to the corresponding author.

## Author Contributions

GD, YS, YC, and TW designed, GD, YS, HP, FH, YC, MW, OB, and SM carried out the experiments. CF and TD provided resources. GD, JJ, YS, YC, SZ, and TW developed methodologies. YS, MC, DD, SZ, and GD prepared and edited the manuscript. All authors contributed to the article and approved the final version.

### Conflict of Interest

The reviewer JS declared a shared affiliation, with no collaboration, with one of the authors TD to the handling editor at the time of the review.

The remaining authors declare that the research was conducted in the absence of any commercial or financial relationships that could be construed as a potential conflict of interest.

## Footnotes

^1^http://www.fao.org/faostat/en/#data/QC
