# Supplementary material for: Investigation of Salt Tolerance Mechanisms Across a Root Developmental Gradient in Almond Rootstocks
Source: Front Plant Sci. 2021 Jan 5;11:595055. doi: 10.3389/fpls.2020.595055 (PMC7813803; doi:10.3389/fpls.2020.595055)
Supplement: Supplementary file 1 [file Data_Sheet_1.pdf]

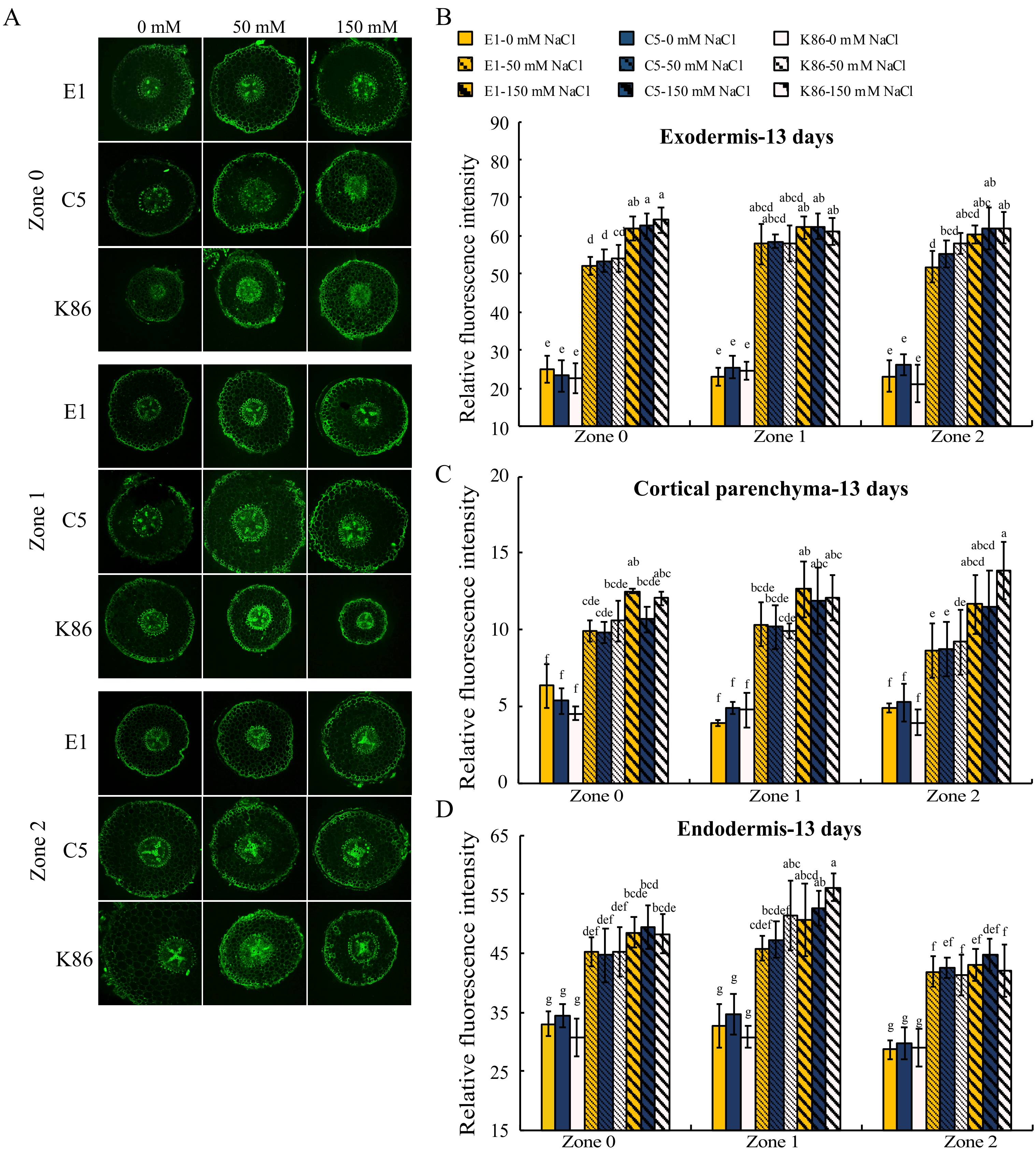

Supplemental Figure. E1 shows lower sodium accumulation under salinity stress compared with C5 and K86. (A) Representative images of CoroNa Green staining in the different root developmental zones in E1, C5 and K86 treated with 0mM, 50 mM, 150 mM NaCl for 13 days. Scale Bars, 100μm. (B) Quantification of sodium signal in the exodermis, (C) cortical parenchyma, and (D) endodermis shows lower fluorescent signal, etc. Values are means ± SE (n = 15). Different lowercase letters indicate significant differences at $P < 0.05$, three-way ANOVA.

Supplemental Table 1. Primer sequences used in the RT-qPCR.

Gene	Primer direction	5'- 3' sequence
ACTIN2	sense	ATCATGAAGTGTGATGTGGATAT
ACTIN2	antisense	ACCTTAATCTTCATGCTGCTTGG
NHX1	sense	ACGTTGCAGGTGCTCAAT
NHX1	antisense	CACCACAGATGTAGCATCGTTA
NHX2	sense	TGCTAACCACTGGAGGGA
NHX2	antisense	TGGAACCCGGCATTGAAA
SOS1	sense	GGCATTCGAATTTGGGCAAA
SOS1	antisense	TTTGTACCATACACCTCTTTATTTGG
SOS2	sense	GACCTGAAGCCCGAGAATC
SOS2	antisense	AAGACCAACTCCTTGTTCAGG
SOS3	sense	GAAACACCCTTTACAGTGAATGAAG
SOS3	antisense	GAAATTCTTCCTTGTGGATAAGCC
HKT1	sense	TCTTTCATGCCAACCCTTTCT
HKT1	antisense	GCCTAGTAGTTCTTGGCTTTGT
SAL1	sense	CTCTCCTCTGCCAGAAGGTA
SAL1	antisense	CAAGAGCCTGTGAACCGTAA
P5CS1	sense	CGCATCGTTCTCAAGGTTG
P5CS1	antisense	TTCAACTCCTTGAGCTGCTC
ASFT1	sense	AGCCCACCACTGCCAAAA
ASFT1	antisense	CATCTTGAACCAGCCCAACT
KCS1	sense	GTGGGAGATGGATTGAGTGAAG
KCS1	antisense	GCTGGAGTGTAGTGGTAGAAGCA
C3H	sense	ACAAGAGTGGTGGTGCCAAG
C3H	antisense	ACGGTTATGGCTGTGGTGTC
